# Course of the extensor pollicis longus tendon considering the different functional positions of the wrist and the first ray—an anatomical study

**DOI:** 10.1007/s10354-024-01052-w

**Published:** 2024-08-05

**Authors:** Peter Grechenig, Theresa di Vora, Amir Koutp, Alexandros Andrianakis, Paul Puchwein, Gloria Hohenberger

**Affiliations:** 1 Department of Orthopaedics and Trauma Surgery, Medical University of Graz, Graz, Austria; 2 Medical University of Graz, Graz, Austria; 3 Department of Otorhinolaryngology, Head and Neck Surgery, Medical University of Graz, Graz, Austria; 4 Department of Trauma Surgery, State Hospital Feldbach-Fürstenfeld, Feldbach, Austria

**Keywords:** Screw protrusion, Distal radius fracture, Tendon rupture, Treatment, Hand injury

## Abstract

**Background:**

The aim of this anatomical study was to evaluate the course of the extensor pollicis longus (EPL) tendon, its positional relationship to adjacent structures, and the resulting clinical relevance under consideration of various functional positions.

**Materials and methods:**

Twenty upper extremities from ten adult human cadavers embalmed using Thiel’s method were included in this study. The greatest possible movement/slippage of the EPL tendon, the angle at which the tendon wraps around Lister’s tubercle, and its course across the extensor carpi radialis longus and brevis (ECRL and ECRB) were recorded and defined in all functional positions.

**Results:**

Our findings demonstrate a high range of motion of the tendon in relation to clinically relevant structures.

**Conclusion:**

Understanding the anatomical course of the EPL tendon, its potential extent of movement, and its resulting positional changes is essential for the diagnosis and surgical treatment of patients with complaints or injuries in the dorsoradial wrist region.

## Introduction

The extensor tendons in the radiocarpal region are essential landmarks during various surgical procedures. Comprehending the anatomy and trajectory of these tendons is clinically crucial for assessing the proximity and positional relationship to inserted implants. Rupture of the EPL tendon is a recognized complication following distal radius fractures. Both conservative and surgical treatments of distal radius fractures can also result in percutaneous rupture of the EPL tendon [1] (e.g., insertion of Kirschner wires during the treatment of radius fractures or carpal injuries, intramedullary nailing of forearm fractures in children [where the implant entry point is initially near Lister’s tubercle], and irritation of tendons by dorsally protruding implants after treating radius fractures through a volar approach) [2]. Additionally, tendon rupture is a clinical consequence of rheumatoid arthritis. With an incidence of approximately 6%, EPL tendon rupture is infrequent but clinically significant [3]. Surgical reconstruction is generally performed using an extensor indicis proprius transfer [4].

Standard dorsal, dorsoradial, and volar approaches are employed for the surgical management of distal radius fractures [5]. Simple or combined approaches are selected based on the type of fracture, the intended surgical procedure, and the osteosynthesis material.

The palpable Lister’s tubercle and the tendon path across the radiocarpal joint, along with the adjacent palpable soft spot, function as surgical landmarks during joint puncture, wrist arthroscopy, and surgical access to the distal radius and carpus. Potential anatomical variations and shifts in tendon position due to different functional positions of the radiocarpal joint and thumb are pertinent for daily clinical practice [6, 7].

Additionally, the tendon path in relation to bony landmarks on sectional imaging is crucial. During surgical wound treatment involving suspected nerve and tendon injury, the functional position of the hand at the time of the accident must always be considered, and surgical exploration should account for this.

The course of the EPL tendon, which encircles the Lister’s tubercle in a radial–distal direction, is particularly significant. While the tendons of other forearm muscles follow an almost straight path distally, the long thumb extensor tendon deviates at an angle of 30–40° from the median in the radial–distal direction, crossing over the underlying tendons of the ECRL and the ECRB [8].

Another point of interest is that the position relative to adjacent structures and bony landmarks changes with different functional positions of the hand (wrist and thumb). This is highly relevant during clinical and imaging diagnostics of chronic complaints in the radiocarpal region as well as during surgical treatments.

The aim of the following anatomical study was to evaluate the course of the EPL tendon and its positional relationship to adjacent structures, while considering various functional positions and the resulting clinical relevance.

## Anatomy

**Fig. 1:**
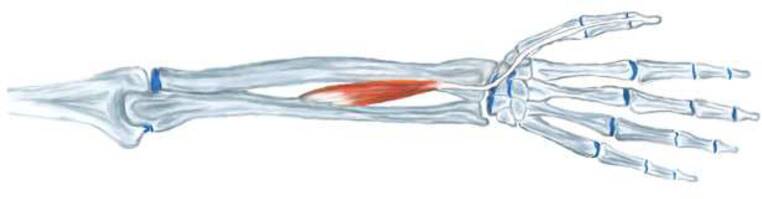
Illustration of the course of the extensor pollicis longus tendon

In the forearm, the tendon travels primarily in a straight path. Around Lister’s tubercle, it is firmly anchored in the third tendon compartment. However, upon exiting this compartment, significant mobility is observed depending on the functional position of the hand and thumb ([7]; Fig. 1).

## Materials and methods

### Specimens

Twenty upper extremities from ten adult human cadavers embalmed using Thiel’s method were utilized for this study [9]. This special embalming technique creates a life-like model by preserving the original tissue color, consistency, and degree of transparency. None of the specimens exhibited signs of malformations or prior interventions in the area of interest.

### Dissection

After removing the skin, subcutaneous tissue, and forearm fascia, the muscle structures were meticulously examined. Additionally, the courses of the tendons were dissected with care to avoid manipulating the extensor retinaculum.

### Measurements

**Fig. 2:**
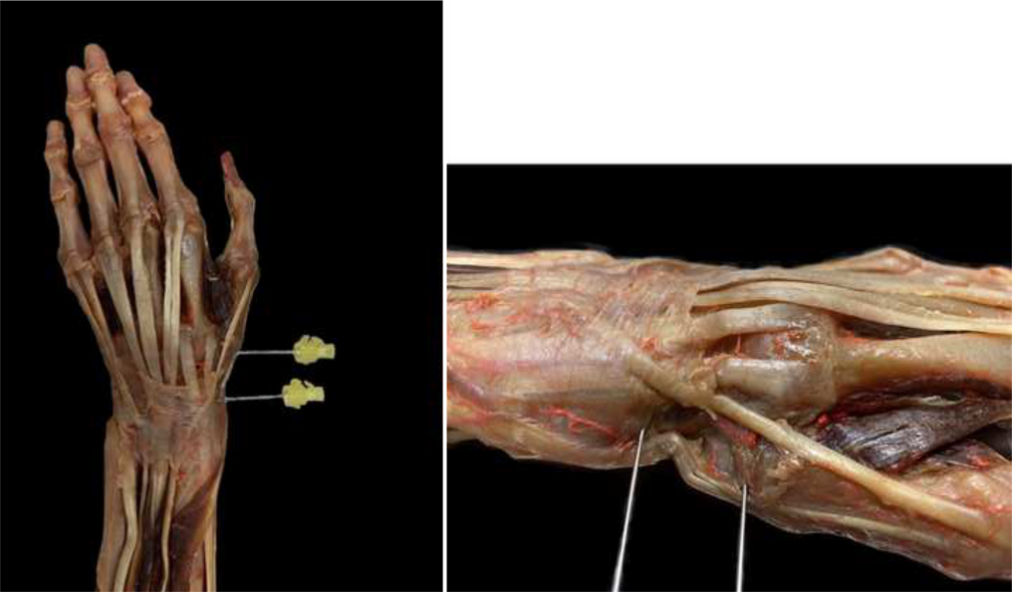
The pins illustrate reference points 1 and 2 (reference point 1: distance between the center of the first carpometacarpal joint and the center of the EPL tendon; reference point 2: distance from the most prominent tip of the radial styloid process to the EPL tendon center)

In the initial series of studies, functional positions that resulted in the maximum possible extension of tendon movement were identified. The distance between the center of the first carpometacarpal joint and the center of the EPL tendon (reference point 1), as well as that from the most prominent tip of the radial styloid process to the center of the EPL tendon (reference point 2), were used as reference points (Fig. 2).

**Fig. 3:**
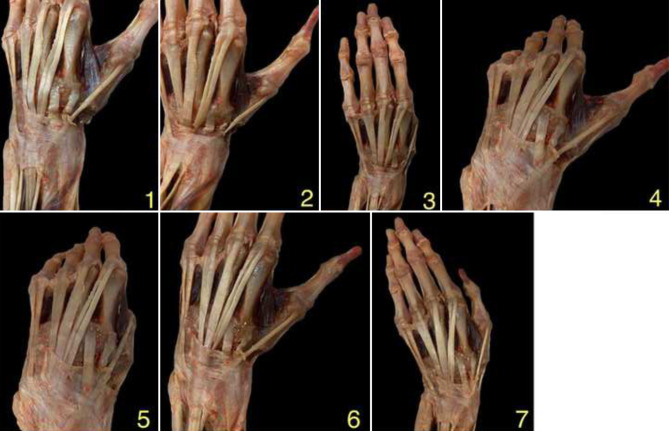
Various positions that lead to the greatest possible range of motion of the EPL tendon. *1* wrist in neutral position, thumb in extension; *2* wrist in extension, thumb in extension; *3* wrist in extension, thumb in opposition; *4* wrist in flexion, thumb in extension; *5* wrist in flexion, thumb in opposition; *6* wrist in radialduction, thumb in extension; *7* wrist in ulnarduction, thumb in adduction

After prior evaluation of the maximum extents of the EPL tendon, the following functional positions of the wrist and thumb were measured (Fig. 3):

1. Wrist in neutral position, thumb in extension
2. Wrist in extension, thumb in extension
3. Wrist in extension, thumb in opposition
4. Wrist in flexion, thumb in extension
5. Wrist in flexion, thumb in opposition
6. Wrist in radialduction, thumb in extension
7. Wrist in ulnarduction, thumb in adduction

**Fig. 4:**
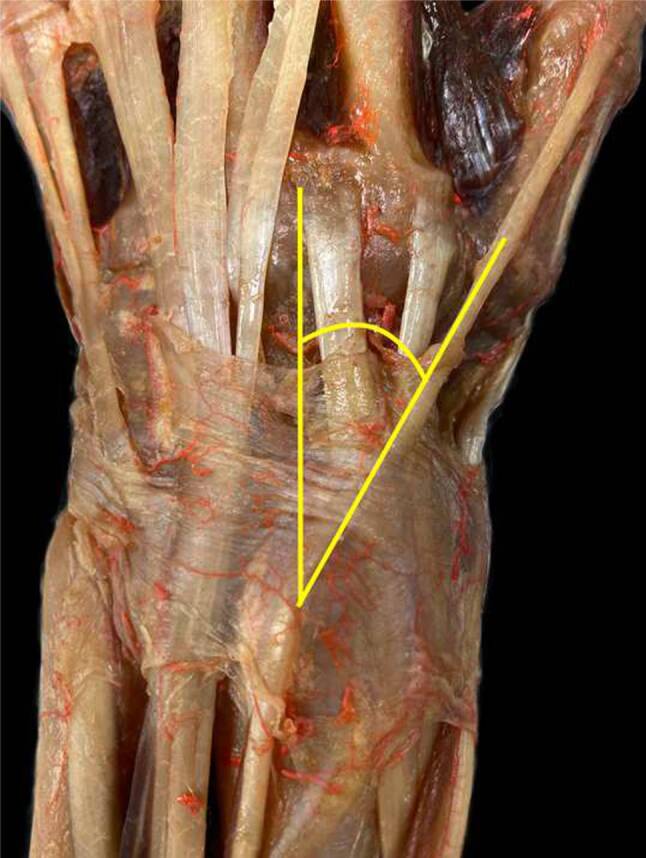
Illustrates the angle at which the extensor pollicis longus tendon wraps around the lister tubercle

The angle at which the EPL tendon wraps around Lister’s tubercle and courses across the ECRL and ECRB was also measured in all functional positions using an angle measurement (Fig. 4). In this series, the functional positions leading to the maximum and minimum possible tendon movement over the ECRL and ECRB were identified.

Maximum angle:

- Wrist in extension, thumb in abduction
- Wrist in radial deviation, thumb in abduction

Minimum angle:

- Wrist in ulnar deviation, thumb in adduction
- Wrist in ulnar deviation, thumb in opposition

During this study, tendon slippage was also assessed. The portion of the EPL tendon distal to the extensor retinaculum was marked with a cannula and measured in all functional positions from the most prominent point of Lister’s tubercle.

The greatest movement of the EPL tendon away from Lister’s tubercle was observed in the following functional positions:

- Wrist in neutral position, thumb in opposition
- Wrist in extension, thumb in opposition
- Wrist in radial deviation, thumb in opposition

The maximal proximal movement of the EPL tendon toward Lister’s tubercle was observed in the following functional positions:

- Wrist in flexion, thumb in extension
- Wrist in ulnar deviation, thumb in extension

## Results

### Range of movement

The range of motion for the thumb ray with the wrist in a neutral position and the thumb extended averaged 15.8 mm from reference point (RP) 1 and 14.4 mm from RP2. When the wrist and thumb were in an extended position, the measurements were 19.76 mm for RP1 and 21.23 mm for RP2.

For the wrist in extension and the thumb in opposition, the values were 19.62 mm for RP1 and 20.55 mm for RP2. When the wrist was flexed and the thumb was extended, the range of motion was 15.14 mm for RP1 and 10.55 mm for RP2. In opposition, the measurements were 13.79 mm for RP1 and 10.33 mm for RP2.

With the wrist in radial deviation and the thumb in extension, the values were 15.9 mm for RP1 and 14.94 mm for RP2. For the wrist in ulnar deviation and the thumb in adduction, the values were 14.64 mm for RP1 and 17.12 mm for RP2.

### Angle around Lister’s tubercle

The maximum angle was achieved with the wrist in extension and the thumb in abduction (mean: 46°), and with the wrist in radial deviation and the thumb in abduction (mean: 55°).

The minimum angle was observed with the wrist in ulnar deviation and the thumb in adduction (mean: 13.5°), and with the wrist in ulnar deviation and the thumb in opposition (mean: 14°).

### Tendon gliding

For the wrist in a neutral position with the thumb in opposition, the distal tendon movement was 10.41 mm. With the wrist in extension and the thumb in opposition, the mean value was 10.64 mm, and for the wrist in radial deviation and the thumb in opposition, the mean value was 10.41 mm.

With the wrist in flexion and the thumb in extension, the values were −6.34 mm, and with the wrist in ulnar deviation and the thumb in extension, the mean value was −7.71 mm.

## Discussion

As surgical procedures increasingly utilize minimally invasive techniques, reduction maneuvers are executed through small incisions, fragments are stabilized, arthroscopically assisted methods are employed, and implants are inserted. Consequently, understanding topographic anatomy, particularly potential anatomical variants, becomes even more crucial [10].

Due to the extensive functional range of the thumb ray combined with the functional position of the wrist, there is significant variation and range of movement of the thumb extensor tendon in terms of its radiocarpal positioning.

The localization of this tendon in diagnostic imaging (MRI, ultrasound) relative to bony landmarks and the radiocarpal joint space is thus highly variable. In the event of injuries in this region, the visible wound (skin laceration) and a potential underlying tendon injury may be distant from each other; therefore, precise knowledge of the course of the EPL and of variants thereof is essential.

During hand movements in the sagittal plane (flexion and extension), it should be noted that the tendon deviates from the frontal plane in relation to the wrist. Additionally, when the muscle contracts (active muscle tension), the tendon protrudes from the metacarpal bone, whereas in the wrist region, tendons are secured in their respective compartments by the extensor retinaculum.

In the surgical management of symptomatic chronic EPL tendon rupture, understanding the anatomical course is fundamental to treatment. The procedure involves an extensor indicis transfer, where the thumb extensor tendon is replaced by a segment of the extensor indicis proprius tendon [11].

Sato and colleagues investigated the incidence of EPL tendon rupture following plate osteosynthesis of the radius through a literature review. EPL rupture was reported in 1.8% of cases (2/114) in the study by Arora et al., in 1.9% (7/335) in Casaletto et al., and in 0.3% (2/665) of cases in the study by Esenwein et al. [12–15].

Naito et al. identified tendon sheath fibrillation in 52% and tendon laceration in 36% of cases in their study of 25 distal radius fractures with dorsal cortical fragments. Similarly, in cases of chronic complaints (pain, tendon crepitus) following osteosynthesis, the positional relationship to the EPL tendon must be evaluated in terms of relevant functional positions (activities of daily living). For instance, the end of a protruding intramedullary wire (these wires are inserted either in the region of Lister’s tubercle or radially near the styloid and capped above the bone level for future removal) can be placed at a safe distance from the tendon in a neutral position. However, in certain functional positions, contact between the tendon and the wire may occur, potentially leading to tendinopathy [16, 17].

## Conclusion

In summary, understanding the anatomical course of the extensor pollicis longus tendon, along with the potential range of its movement and resulting positional changes, is essential for accurately diagnosing and surgically treating patients with complaints or injuries in the dorsoradial wrist region.

## Data Availability

The datasets used and/or analyzed during the current study are available from the corresponding author upon reasonable request.
